# Silencing of miR-370 in Human Cholangiocarcinoma by Allelic Loss and Interleukin-6 Induced Maternal to Paternal Epigenotype Switch

**DOI:** 10.1371/journal.pone.0045606

**Published:** 2012-10-22

**Authors:** Fangmei An, Sumitaka Yamanaka, Sarah Allen, Lewis R. Roberts, Gregory J. Gores, Timothy M. Pawlik, Qing Xie, Masaharu Ishida, Esteban Mezey, Anne C. Ferguson-Smith, Yuriko Mori, Florin M. Selaru

**Affiliations:** 1 Division of Gastroenterology and Hepatology, Johns Hopkins Hospital, Baltimore, Maryland, United States of America; 2 Department of Infectious Diseases, Ruijin Hospital, Shanghai Jiao Tong University School of Medicine, Shanghai, People's Republic of China; 3 Department of Gastroenterology, Wuxi People's Hospital Affiliated to Nanjing Medical University, Wuxi, Jiangsu, China; 4 Department of Physiology, Development and Neuroscience, University of Cambridge, Cambridge, United Kingdom; 5 Divisions of Gastroenterology and Hepatology, College of Medicine, Mayo Clinic, Rochester, Minnesota, United States of America; University of North Carolina School of Medicine, United States of America

## Abstract

Cholangiocarcinoma (CCA) is a highly lethal malignant tumor arising from the biliary tract epithelium. Interleukin-6 (IL-6) is a major mediator of inflammation and contributor to carcinogenesis within the biliary tree. Previous studies suggested that enforced IL-6 contributes to cholangiocarcinogenesis through hypermethylation of several genes implicated in CCA. However, the precise mechanisms of IL-6 effects in CCA remain unclear. We now demonstrate that microRNA (miR)-370 is underexpressed in a large cohort of human CCA *vs.* normal liver tissues. In addition, we show that IL-6 induces a time-dependent silencing of miR-370. In addition, demethylation of CCA cells results in upregulation of miR-370. Furthermore, we demonstrate that miR-370 is imprinted, and that the Intergenic Differentially Methylated Region (IG-DMR) responsible for imprinting regulation of this genomic locus is hypermethylated in response to IL-6 treatment. In addition, the IG-DMR is hypermethylated in human CCA specimens compared to normal matched controls, in the same location as the IL-6 induced hypermethylation. Finally, miR-370 was found to regulate WNT10B in luciferase as well as western blotting experiments. Our data indicate that the paternal allele of miR-370 is normally silenced through genomic imprinting and that the overexpression of IL-6 in CCA effectively suppresses the expression of miR-370 from the maternal allele, lending support to the theory that miR-370 silencing in human CCA follows a classic two-hit mechanism.

## Introduction

Cholangiocarcinoma (CCA) is an aggressive tumor of the biliary tract [1]. CCAs are usually diagnosed late in their progression, and the patient survival is usually measured in months [2]. Molecular characterization of CCAs further suggested that inflammation and cholestasis, through modulation of genes involved in DNA damage repair, promote cancer development [1]. IL-6 is a recognized mitogen and survival factor in human CCA and can contribute to tumor pathogenesis or progression [3]. Therefore, it appears that elucidation of pathways downstream of IL-6 merits further investigation.

DNA methylation refers to the addition of a methyl group to a cytosine residue in a sequence of DNA often resulting in silencing of gene expression [4]. Aberrant DNA methylation has been implicated in CCA [5]. Epigenetic regulation of tumor suppressor genes by DNA hypermethylation has been recognized as an important mechanism in tumorigenesis [6]. Several studies found that IL-6 contributes to the growth of CCA cells by inducing aberrant promoter DNA methylation [7], [8]. Interestingly, methylation is also an important mechanism for regulation of imprinting [9]. Genomic imprinting represents a form of epigenetic control of gene expression in which one allele of a gene is preferentially expressed according to the parent-of-origin [10]. The term “imprinting” was coined in 1960 [11], however, it was not until 1991 that the first mammaliam imprinted genes were identified [12], [13], [14]. Imprinting demonstrated the importance of epigenetic control of gene expression both in normal circumstances (embryologic development) as well as in pathological conditions, such as cancer [9]. Loss of imprinting was first linked to cancer in 1993, when overexpression to 2-fold of Insulin-like growth factor 2 (Igf2) was identified in Wilms' tumor [15], [16]. The same alteration was later identified in Beckwith–Wiedemann syndrome (BWS) [17] and other neoplasias, such as lung cancer and chronic myelogenous leukemia [18], [19]. Although several studies implicated dysregulated imprinting in hepatocellular carcinoma, there are no studies to date to link imprinting to CCA [20], [21].

The DLK1-DIO3 imprinted domain is located on human chromosome 14q32.2 and is associated with expression of protein-coding genes from the paternally inherited chromosome (*Delta-like homologue 1* (*DLK1*), *Retrotransposon-like gene 1* (*RTL1/MART1*), and the type 3 deiodinase (*DIO3*)) as well as non-protein coding RNAs from the maternally inherited chromosome including clusters of microRNAs [22]. However, direct verification of the imprinting status of specific non-coding RNAs in cancer has not been performed so far.

We previously reported, based on microRNA arrays, that micro-RNA-370 (miR-370) appears to be downregulated in human cholangiocarcinomas [23]. In addition, it was previously reported that enforcing IL-6 expression in CCA cell lines induces miR-370 downregulation correlating with increased expression of DNA methyltransferase enzyme-1 (DNMT-1) [24]. Although the role of IL-6 in downregulating miR-370 in human specimens continues to be elusive, these data suggest that miR-370 might be an important etiologic factor in CCA. Of note, miR-370 is located within the DLK1-DIO3 domain, but so far there is no data regarding its imprinting status in human CCA specimens.

## Results

### MiR-370 is down regulated and IL-6 is up regulated in human CCA *vs.* normal tissues

**Figure 1:**
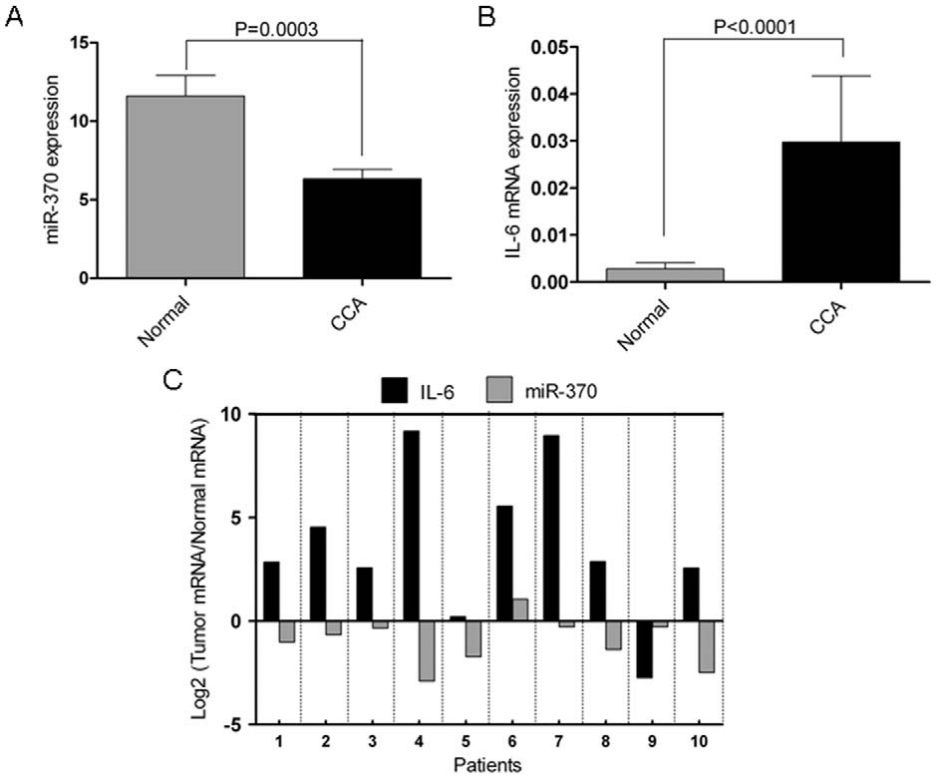
miR-370 and IL-6 are inversely correlated in human CCA. A. miR-370 is downregulated in human CCA *vs.* normal liver tissues. The figure displays the mean and standard deviation of qRT-PCR-measured expression of miR-370 normalized to RNU6B for human CCA and normal liver tissues. **B. IL-6 is upregulated in human CCA** ***vs.*** **normal liver tissues.** The figure displays the mean and standard deviation of qRT-PCR-measured expression of IL-6 mRNA normalized to β-actin for human CCA and normal liver tissues. **C. miR-370 and IL-6 display an inverse relationship in matched human CCA and normal specimens.** Ten pairs of CCA and matched normal liver tissues for which we had expression data for both miR-370 and IL-6 were analyzed. For each CCA specimen, we compared the level of miR-370 and IL-6, respectively, to their corresponding normal specimen. The figure displays the Log2 of the ratio of miR-370 in CCA *vs.* matched normal as well as the Log2 of the ratio of IL-6 in CCA *vs.* matched normal specimen. Since the data is displayed in log space, a positive value on the Y-axis for miR-370 or IL-6 represents overexpression of miR-370 or IL-6, respectively, in a CCA specimen *vs.* matched normal specimen. Conversely, a negative value on the Y-axis represents underexpression of miR-370 or IL-6, respectively, in the CCA specimen *vs.* matched normal specimen. The X-axis displays the 10 pairs of CCA and matched normal specimens.

Our previous miR microarray data performed on a small cohort of CCAs suggested that miR-370 is downregulated in cancers *vs.* normal tissue [23]. However, to date, the expression level of miR-370 has not been validated in a larger cohort of human CCA specimens. Therefore, we assayed the expression of miR-370 in 58 human specimens, including 34 CCAs and 24 normal liver tissues. The average expression of miR-370 *vs.* RNU6B in CCA (6.33) was lower than in normal tissues (14.8-fold, p-value<0.001, unpaired Student's t-test, **Figure 1A**), validating our preliminary observations. We then assayed the expression of IL-6 in the same specimens. We found that IL-6 was statistically significantly upregulated in CCA *vs.* normal tissues (14-fold, p-value<0.0001, unpaired Student's t-test, **Figure 1B**). Based on these data we sought evidence of an inverse relationship between the expression levels of IL-6 and miR-370 in human specimens. Among our specimens, we had 10 pairs of CCA tissues and matched normal liver tissues for which we had expression data for both miR-370 and IL-6. In 8 out of 10 pairs of specimens, IL-6 had higher expression in CCA *vs.* matched normal liver, while miR-370 had lower expression in CCA *vs.* matched normal liver (**Figure 1C**), suggestive of an IL-6 regulatory effect on miR-370 in human CCA specimens.

### IL6 inhibits miR-370 through modulation of DNA methylation levels

**Figure 2:**
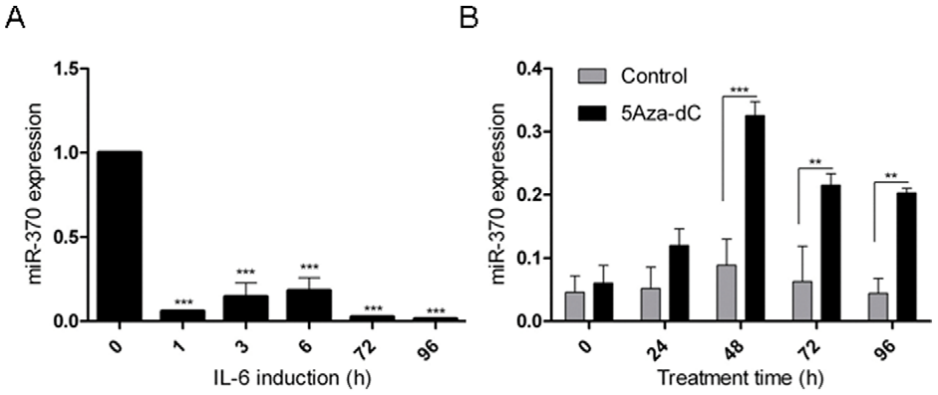
IL-6 and promoter methylation modulate expression of miR-370. A. IL-6 reduces miR-370 expression in HuCCT1 cells. X-axis – duration of IL-6 treatment (in hours). Y-axis – Mean and standard deviation of qRT-PCR-measured expression of miR-370 normalized to RNU6B. **B. Treatment with the demethylating agent 5Aza-dC upregulates miR-370 expression in HuCCT1 cells.** Black column - 5Aza-dC-treated cells, gray column – negative control. X-axis –the duration of 5 Aza-dC treatment (hours). Y-axis – Mean and standard deviation of qRT-PCR-measured expression of miR-370 normalized to RNU6B. Data represents the mean value of three independent experiments, **P<0.01, ***P<0.001.

To test this hypothesis and confirm *in vitro* that IL-6 downregulates miR-370, we treated human intrahepatic CCA cells, HuCCT1, with IL-6 and assayed miR-370. The expression level of miR-370 starts decreasing at 1 hour and appears to be maximally suppressed starting with 72 hours post treatment (p-value<0.0001, One-way ANOVA, **Figure 2A**), in accord with data in other CCA cell lines [24]. Although the quick suppression of miR-370 within 1 hour of starting the treatment cannot be explained through increased methylation, miR-370 demonstrates maximal suppression starting at 72 hours post treatment. The early effects of IL-6 onto miR-370 levels could be a result of transcription regulation, as previously suggested by others {Loffler, 2007 #1212}. At 96 hours, the expression of miR-370 is almost zero and this finding could be, at least in part, due to increased methylation. It is possible, therefore, that methylation is acquired secondary to IL-6 treatment and may contribute to the maintenance of repressed gene expression. To test if demethylating agents influence the expression of miR-370, we treated HuCCT1 cells with the demethylating agent 5 Aza-deoxycytidine (5 AZA-dC) and noted, at 48 hours post treatment, a sharp and statistically significant rise in expression level of miR-370 *vs.* negative control (**Figure 2B**). We verified these results in a colorectal adenocarcinoma cell line HCT116 (**Figure S1**). These data suggest that, IL-6 exerts its effects, at least in part, through changes in DNA methylation levels.

### Confirmation of imprinting status of miR-370

**Figure 3:**
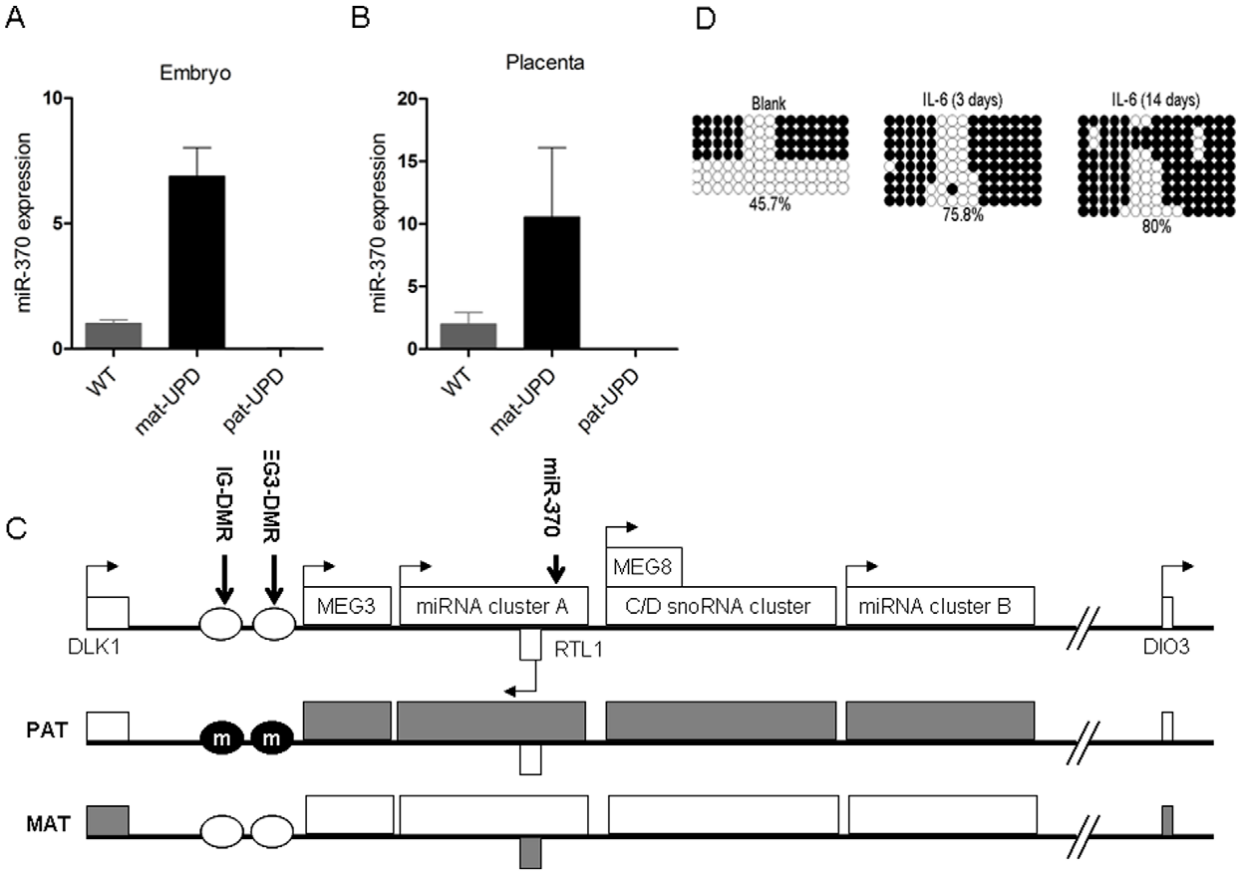
miR-370 is normally imprinted. **A** and **B.** **Maternal, but not paternal, UPD(12) disomy mice express miR-370.** To establish the imprinting status of miR-370, TaqMan qPCR was carried out on wild-type mice and mice with maternal and paternal uniparental disomy for chromosome 12 (WT, mat-UPD and pat-UPD respectively). miR-370 expression is strongly elevated in mat-UPD embryos and is absent from pat-UPD embryos, showing that miR-370 is a maternally expressed imprinted gene. Mean ± SD, *P<0.05, **P<0.01. **C.** **miR-370 is located within the DLK-DIO3 domain.** The genomic map of imprinted DLK-DIO3 control domain is displayed: gray rectangles indicate silenced alleles and the blank rectangles indicate expressed alleles, the paternally (PAT) expressed alleles are DLK1, RTL1 and DIO3. The maternally (MAT) expressed non-coding RNA genes include maternally expressed gene 3 (MEG3), anti-RTL1 (encodes the smaller miR cluster A), maternally expressed gene 8 (MEG8) (encodes small nucleolar RNA (snoRNA) cluster) and the larger miR cluster. IG-DMR and MEG3-DMR are methylated on the paternal chromosome indicated by the encircled letter m. miR-370 is a member of the cluster A of miRs and located within the DLK1-DIO3 domain. **D.** **IL-6 induces gain of methylation at IG-DMR-CG6 in HuCCT1 cells.** Each line indicates a single clone (either of maternal, or paternal origin), and each circle denotes the cytosine of a CpG site; filled and open circles represent methylated and unmethylated cytosines, respectively. The number at the bottom indicates the percentage of methylated cytosines.

The inverse correlation between IL-6 and miR-370 in human CCA specimens and matched normal livers, as well as the fact that IL-6 induces miR-370 suppression *in vitro* while demethylation induces miR-370 overexpression suggests a potential pathway where overexpression of IL-6 in human CCA induces higher levels of DNA methylation which, in turn, induces lower levels of miR-370. Interestingly, the genomic locus of miR-370 is situated within the DLK1-DIO3 imprinted domain. Therefore, we hypothesized that the paternal allele of miR-370 is repressed by imprinting. We further hypothesized that miR-370, normally expressed only from the maternal allele, is suppressed by IL-6 via hypermethylation-mediated transcriptional repression. To establish if the prediction of miR-370 imprinting based on the location of its genomic locus is translated to tissues, we assayed the level of miR-370 in embryos with alterations in the parental origin of mouse chromosome 12 (uniparental disomy mice) which would be predicted to express either a double dose (maternal uniparental disomy) or no miR-370 (paternal uniparental disomy) if the gene is imprinted. These mice were created by intercrossing mice doubly heterozygous for Robertsonian translocations with monobrachial homology for chromosome 12 as described previously [25]. As **Figure 3A and B** demonstrate, the expression of miR-370 in wild type control littermates is, as expected, approximately half the maternal disomy levels, while the expression of miR-370 in paternal disomy tissues is non-existent.

### IL-6 induces hypermethylation of the DLK1-MEG3 intergenic differentially methylated region (IG-DMR)

The imprinting status of the DLK1-DIO3 domain is controlled, at least in part, by the germline-derived primary DLK1-MEG3 intergenic differentially methylated region (IG-DMR) which regulates the postfertilization-derived secondary MEG3-DMR which is also an important regulator of the locus [26], [27] (**Figure 3C**). Within the IG-DMR, there are CG-rich sequences, among which is IG-DMR-CG6 region, whose abnormal methylation was shown previously to be consistent with human uniparental disomy (upd) (chromosome 14) paternal/maternal phenotypes [28]. Therefore, we analyzed the methylation status of IG-DMR-CG6 both at baseline and in response to IL-6 treatment. We performed bisulfite sequencing of IG-DMR-CG6 after treatment with IL-6, and found gain of methylation by day 3 that progressed to higher levels at day 14 (**Figure 3D**). These data suggest that IL-6 stimulation can induce hypermethylation at IG-DMR-CG6, which, in turn, may result in aberrant behavior of the maternally inherited chromosome at the DLK1-DIO3 domain.

### IG-DMR-CG6 is hypermethylated in human CCA *vs.* normal matched specimens

**Figure 4:**
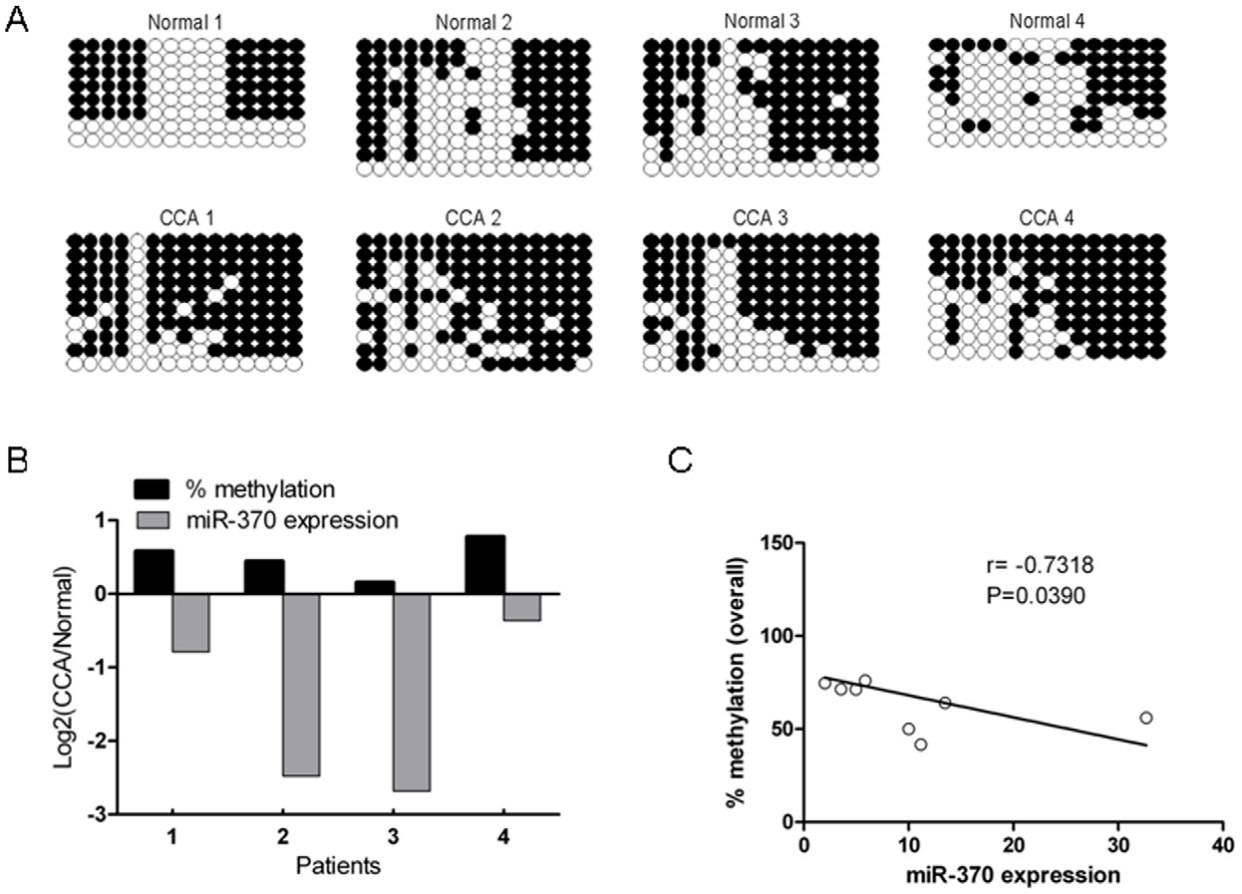
Methylation of IG-DMR-CG6 influences miR-370 expression. **A.** **IG-DMR-CG6 is hypermethylated in human CCAs** ***vs.*** **matched normal tissues.** Each line indicates a single clone, and each circle denotes the cytosine of a CpG site; filled and open circles represent methylated and unmethylated cytosines, respectively. **B.** **miR-370 expression is inversely related to methylation at IG-DMR-CG6 in human liver specimens.** X-axis - methylation rate, and miR-370 expression, Y-axis - human tissues. Percent methylation and miR-370 expression are expressed as a log2 of the ratio between the cancer and matched normal specimen. Since the data is expressed in logarithmic space, a positive value (as in the values for methylation for all 4 specimens) signifies more methylation in cancer *vs.* matched normal tissue. A negative value (as in the values for miR-370 for all 4 specimens) signifies a lower expression in cancer *vs.* matched normal specimen. These data demonstrates that for all 4 pairs of specimens, an increased in the methylation at IG-DMR-CG6 in cancer *vs.* matched normal is accompanied by a lower level of miR-370 in cancer *vs.* matched normal specimen. **C.** **The expression of miR-370 is inversely correlated with level of DNA methylation.** X-axis- miR-370 expression, Y-axis- overall methylation rate (%). r-correlation coefficient, p- P value.

To investigate if the *in vitro* data demonstrating increased hypermethylation of IG-DMR-CG6 in response to IL-6 was evident in human specimens, we performed bisulfite sequencing of the IG-DMR-CG6 on human CCAs. Since our specimens were not microdissected, we chose the top 4 CCA specimens that displayed a high epithelial component (>80%) to diminish the influence of IG-DMR-CG6 methylation level from intervening fibroblasts. We found that in all 4 cases, the methylation level of IG-DMR-CG6 was consistently higher in cancer *vs.* matched normal liver (**Figure 4A**). Of note, in all these cases, the level of miR-370 expression was inversely correlated with the level of IG-DMR-CG6 methylation. As **Figure 4B** shows, the percent methylation was higher in cancer *vs.* matched normal tissues (black bars towards the positive side of Y axis) and the expression of miR-370 was less in cancer *vs.* matched normal tissues (the figure displays the logarithmic (base 2) value of the ratio between expression of miR-370 in cancer *vs.* normal tissues, therefore the dark grey bars point towards the negative side of the Y axis). Next, we plotted all 8 tissues (4 cancer and their matched 4 normal specimens) in terms of percent methylation (Y-axis in **Figure 4C**) and miR-370 expression (X-axis in **Figure 4C**) and noted a statistically significant correlation between percent methylation and miR-370 expression. These data suggest that hypermethylation of IG-DMR-CG6 in human CCA contributes to diminishing levels of miR-370.

### miR-370 locus displays allelic loss in a subset of human CCA *vs.* matched normal specimens

**Figure 5:**
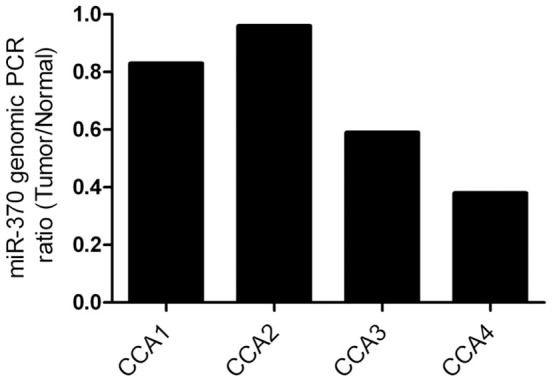
Two of the four CCA tissues displayed LOH at miR-370 locus *vs.* matched normal tissues. For each CCA specimen, we calculated the ratio between the level of miR-370 DNA in cancer *vs.* matched normal specimen. This ratio is displayed on the Y-axis. A ratio close to 1 (as shown for CCA1 and CCA2) signifies no LOH while a ratio close to 0.5 (as in CCA3 and CCA4) suggests LOH at miR-370 in CCA *vs.* matched normal tissue.

To investigate if the lower expression of miR-370 in human CCA *vs.* matched normal can be solely explained by the observed increased methylation at IG-DMR-CG6, we performed PCR on genomic DNA of the genomic locus of miR-370. For each CCA specimen, we compared the normalized level of miR-370 DNA to their corresponding normal specimen. In this fashion, we were able to consider LOH at the miR-370 genomic locus. For these analyses, we employed the same 4 pairs of CCA-normal tissues as above, since these cancers had an epithelial component of at least 80%, to minimize the contribution of intervening fibroblasts. As **Figure 5** shows, in specimens CCA1 and CCA2, the ratio of miR-370 DNA in tumor *vs.* normal is 0.83 and 0.96, respectively, which suggests that in these specimens, there is no LOH at miR-370 locus. In contrast, specimens CCA3 and CCA4 demonstrated a ratio of 0.59 and 0.38, respectively, suggesting that the amount of genomic DNA in tumors is approximately half the level in matching normal specimens. Thus, genomic locus of miR-370 appears to display LOH in 2 out of the 4 specimens tested.

### miR-370 displays cell growth suppressive effects

**Figure 6:**
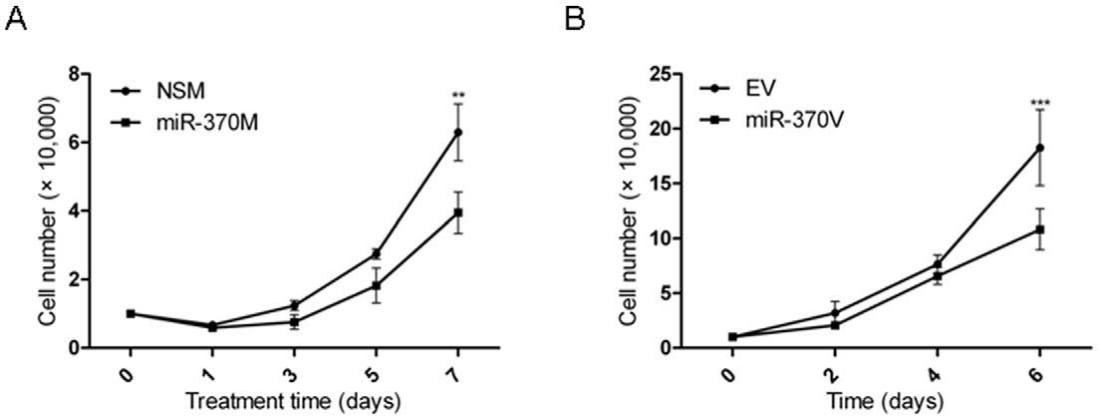
miR-370 induces growth retardation. **A.** **HuCCT1 malignant cholangiocytes display decreased growth upon reinforcement of miR-370 expression.** X-axis – HuCCT1 cells counted at day 1, 3, 5 and 7 after transfection with miR-370M, or NSM, respectively. Y-axis – cell counts ×10^4^ of HuCCT1 cells transfected with miR-370 mimic (miR-370M, black squares) or NSM (black circles). **B.** **HuCCT1 malignant cholangiocytes display decreased growth upon modest miR-370 upregulation through infection with MIEG3-miR-370.** X-axis – HuCCT1 cells counted at days 2, 4 and 6 after plating of MIEG3-miR-370 (miR-370V) HuCCT1 cells or MIEG3-EV (EV) HuCCT1 cells, respectively. Y-axis – cell counts ×10^4^ of miR-370V HuCCT1 cells (black circles) or the EV HuCCT1 cells (black squares). For every treatment and every time point, the data presented is the average of 5 independently counted wells. Mean ± SD, **P<0.01, ***P<0.001.

To assess if miR-370 has tumor suppressive effects on CCA cells, miR-370 mimic or non-specific mimic (NSM) were transfected into HuCCT1 cells. Exogenous miR-370 induced a statistically significant decrease in growth (**Figure 6A**). The growth-suppressive effect of miR-370 overexpression was also confirmed in a dicer-defective cancer cell line, HCT116 Dicer(-) (**Figure S2**).

**Figure 7:**
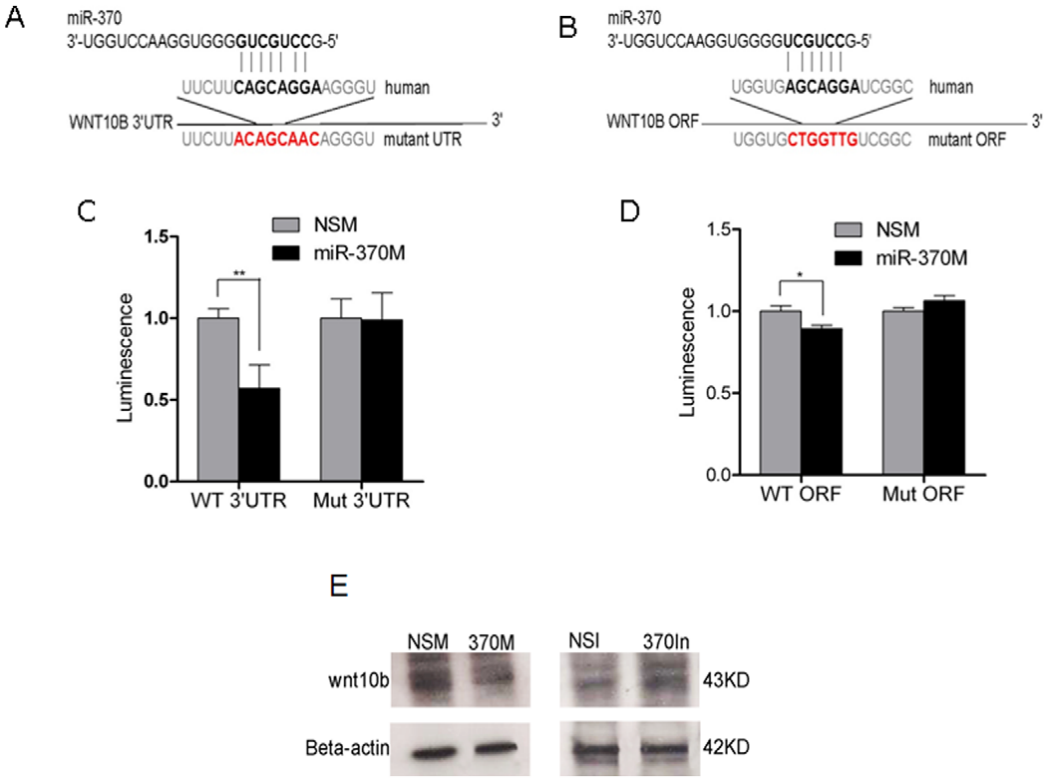
miR-370 modulates the expression of WNT10B. **A and B.** **WNT10B is a putative target of miR-370.** The seed of miR-370 displays complementarity to position 668–674 of WNT10B 3′UTR (**A**) as well as to position 458–464 of WNT10B ORF (**B**) in bold. Mutated nucleotides are shown in red. **C and D.** **miR-370 binds directly to WNT10B.** PGL4 luciferase reporter plasmids containing wild-type WNT10B 3′UTR (C) or open reading frame (ORF) (D) were cotransfected with miR-370 mimic (miR-370M) or non-specific mimic (NSM), respectively. The PGL4 plasmids containing mutant target site of the WNT10B 3′UTR or ORF were also cotransfected with miR-370M or NSM. The firefly luciferase activity was normalized to the Renilla luciferase activity for each sample. Data represents the mean value of three independent experiments. Mean ± SD. *P<0.05, **P<0.01. **E.** **WNT10B protein changes upon miR-370 manipulation.** Equal protein loading was performed, as shown by β-actin. NSM – non-specifici mimic; miR-370M – miR-370 mimic; NSI – non specific inhibitor; miR-370In – miR-370 inhibitor.

To identify the pathway by which miR-370 exerts its tumor-suppressive effects, we sought mRNA targets of miR-370. First, we performed an *in silico* complementarity search, by employing *TargetScan* (http://www.targetscan.org/). Wingless-type MMTV integration site family, member 10B (WNT10B) was predicted as a target of miR-370 (**Figure 7A**). In addition, we identified another miR-370 putative binding site in the ORF of WNT10B (**Figure 7B** **)**. First, we assessed the change in the mRNA level of WNT10B in response to miR-370 level. To this end, we performed RT-PCR for WNT10B on HuCCT1 cells transduced with an empty vector (HuCCT1-EV) and on HuCCT1 cells transduced with miR-370 (HuCCT1-370V). We found that WNT10B mRNA level is decreased with approximately 40% in HuCCT1-370V cells *vs.* HuCCT1-EV cells (**Figure S3**). We next assessed a potential direct interaction between miR-370 and WNT10B *in vitro* using HuCCT1 cells that were transfected with luciferase reporter plasmids containing the wild-type WNT10B mRNA 3′UTR as well as miR-370M or NSM. Luciferase activity in the cells co-transfected with miR-370 mimic was down-regulated by ∼42% (P = 0.001, unpaired Student's t-test), compared with cells transfected with NSM. The effect was rescued when, instead of the WNT10B wild type sequence, a mutated sequence was employed (**Figure 7C**). Similar experiments were conducted to test the direct interaction between miR-370 and the binding site located in the ORF of WNT10B. miR-370 induced ∼10% decreased expression of the ORF (P = 0.018, unpaired Student's t-test), an effect that was lost upon mutating the miR-370 binding site (**Figure 7D**). The effects of miR-370 on the ORF of WNT10B may be biologically less important than the effects on the 3′UTR, however, these effects were statistically significant. These data confirmed that miR-370 can bind directly to both the 3′UTR and the ORF of WNT10B in human cholangiocarcinoma cells. Finally, to confirm that miR-370 has a significant effect on the protein level of WNT10B, we performed western-blotting on proteins extracted from HuCCT1 cells treated with NSM, miR-370, NSI or miR-370 inhibitor respectively. We found that enforced overexpression of miR-370 reduced WNT10B and downregulation of miR-370 resulted in increased WNT10B (**Figure 7E**). These data suggest that WNT10B is a direct target of miR-370.

The transfection of miR-370 results in non-physiologically high levels of miR-370 (**Figure S4A**). In order to confirm the suppressive effects of miR-370 on CCA cells at a physiological level, we inserted miR-370 into a retrovirus, MSCV-IRES-Enhanced-GFP-3 (MIEG3). Cells infected with MIEG3-miR-370 expressed miR-370 approximately 7.5 fold more than cells infected with MIEG3-EV (empty virus), which is similar to the miR-370 upregulation in normal cholangiocytes (H69) *vs.* malignant cholangiocytes (HuCCT1) (**Figures S4B and S4C**). In spite of this modest upregulation of miR-370, cells infected with MIEG3-miR-370V behaved similarly to cells transfected with miR-370, displaying a statistically significant decreased growth (**Figure 6B**).

## Discussion

Several recent studies identified miR dysregulation in human CCA specimens [23], [29], [30]. Our report now brings evidence that miR-370 is downregulated in human CCA *vs.* normal specimens. IL-6 is a major regulator of CCA growth and it inhibits miR-370 *in vitro* [24]. We now show that matched human CCA and normal specimens display an inverse relation between IL-6- and miR-370 expression, suggestive of *in vivo* interaction. We further confirmed *in vitro* that IL-6 downregulates miR-370 and that Azacytidine treatment increases the expression of miR-370. While IL-6 is known to upregulate DNMT1 in malignant cholangiocytes [24], it was previously unclear what mechanisms are involved downstream of DNMT1 activation.

miR-370 localizes within the imprinted locus DLK1-DIO3 [31]. Therefore, we hypothesized that loss of miR-370 in human CCAs can be, at least in part, explained by a two-hit theory, whereby the first “hit” is the result of normal imprinting, while the second hit is the result of an IL-6 induced maternal to paternal epigenotype switch that causes loss of expression from the usually active allele. Indeed, our data suggest that miR-370 is normally imprinted in post-embryonic life and expressed only from the maternal allele. Furthermore, we show that IL-6 stimulation in CCA cells results in hypermethylation of IG-DMR-CG6, with a similar profile to human CCA specimens. Overwhelming evidence suggests that IL-6 is overexpressed in liver malignancies where multiple downstream pathways were identified [32]. Our report indicates that IL-6 can induce an epigenotype switch at an imprinted locus in human cancers and adds further rationale to IL-6 targeting.

Previous studies advanced the hypothesis that IL-6 induces an increase in DNMT1, which, in turn, will result in DNA hypermethylation [24]. These data are in accord with our study. Two previous studies reported loss of heterozygosity (LOH) at 14q32 for some intrahepatic CCAs [33], [34]. These studies, however, did not test LOH at the miR-370 genomic locus. We now report, although in a small number of CCA specimens, allelic loss of the specific genomic locus of miR-370 in approximately 50% of CCAs. However, it is currently difficult to differentiate between LOH of the maternal or paternal allele. Therefore, we cannot easily discriminate between LOH of the paternal allele – which would be biologically irrelevant given that the paternal allele is normally repressed. Nonetheless, our *in vitro* data strongly suggests that IL-6 is able to hypermethylate IG-DMR-CG6 and silence DLK1-DIO3 in those cases where LOH has not already induced a loss of this genomic area. These data further suggest that the DLK1-DIO3 is a potential “hotspot” in liver malignancies, lost through several, but potentially cooperative mechanisms, including LOH and a maternal to paternal epigenotype switch.

Our study contributes to unraveling miR-370 as an important miR in the pathogenesis of liver cancers. Previous studies identified MAP3K8 as a target of miR-370 [24], and we now report WNT10B as a biologically relevant target in human CCAs. Recent data suggests that miRs act as rheostats of canonical pathways through convergent and coherent actions on multiple targets simultaneously [23]. It is therefore likely that miR-370 also acts on multiple targets and it would be interesting to fully dissect molecular pathways downstream of this miR, although this was not the primary focus of the current study.

Although there are no published studies regarding the role of WNT10B in CCA, studies from other cancers suggest that WNT10B overexpression promotes carcinogenesis. For example, WNT10B overexpression driven by an MMTV promoter was found to direct mammary gland hypermorphic development, transformation and eventually adenocarcinoma development [35]. Other studies identified WNT10B overexpression in ovarian endometrioid carcinoma [36], in approximately 58% of B-cell progenitor acute lymphoblastic leukemia [37] and in neuroblastomas [38]. The only report in hepatocellular carcinoma, demonstrated that overexpression of WNT10B in the presence of fibroblast growth factor (FGF) promotes cancer cell growth [39]. Interestingly, a recent report found that the level of FGF in the gallbladder and biliary tree bile is twice the level in the systemic circulation [40]. Moreover, FGF was reportedly found upregulated in sclerosing areas in Primary Sclerosing Cholangitis (PSC), which are areas where CCA usually develops. The interaction between FGF, WNT10B and cancer growth may prove to be highly relevant to the pathogenesis of inflammation induced carcinogenesis in CCA [41]. Our data, in conjunction with these reports, suggest that WNT10B is an attractive target for further studies in CCA.

In conclusion, we report miR-370 is downregulated in human CCA specimens. In addition, we bring evidence suggesting that miR-370 is normally imprinted. The data shown here lends support to the hypothesis that miR-370 is an important mediator of cholangiocarcinogenesis, and its loss can be explained through physiological imprinting, allelic loss, as well as IL-6 induced maternal to paternal epigenotype switch. We observed the growth suppressive effect of miR-370 overexpression *in vitro*, and effects of miR-370 are exerted, at least in part, through WNT10B inhibition.

## Materials and Methods

### Ethics Statement

The current study was approved by the Johns Hopkins University and Mayo Clinic Institutional Review Board (IRB) protocols. Written informed consent was obtained from all patients.

### Human tissues

The human specimens were obtained at surgery performed at the Johns Hopkins Hospital (JHH, Baltimore, MD, US) and the Mayo Clinic (Rochester, MN, US). The indication for surgery was liver resection for CCA. Matched tumor and normal liver were obtained and confirmed histologically. Tissues were snap frozen upon acquisition and stored in a −80°C freezer till use. Genomic DNA and total RNA were extracted from frozen tissues using DNeasy kit (Cat# 51304; QIAGEN Science, Germantown, MD, US ) and Trizol Reagent (Cat# 15596018; Ambion, Carlsbad, CA, US) respectively, as previously described [42], [43].

### Quantitative real time RT-PCR (qRT-PCR)

qRT-PCR was used to evaluate the expression of miR-370 and IL-6 mRNA. TaqMan miR Assay kits (Applied Biosystems, Foster City, CA, US) were used for miR-370 and normalized to RNU6B. Power SYBR Green PCR Master Mix (Cat# 4367659; Applied Biosystems, Carlsbad, CA, US) was used for IL-6 mRNA qRT-PCR. The expression of IL-6 mRNA was normalized to the expression of β-actin. IL-6 primers were the following: 5′-GGTACATCCTCGACGGCATCT-3′ (forward), and 5′-GTGCCTCTTTGCTGCTTTCA C-3′. β-actin primer sequences were described previously [44]. Relative expression of target RNAs was calculated using the delta Ct method as described previously [30]. All PCR reactions were carried out on the 7900 HT Fast Real-time PCR System (Applied Biosystems, Carlsbad, CA, US) in duplicate.

### Cell culture

Human intrahepatic CCA cells, HuCCT1, were maintained in Dulbelcco's Modified Eagle Media (DMEM) supplemented with 10% fetal calf serum (FCS), 1000 U/mL penicillin/streptomycin (P/S), as previously described [23]. Colorectal adenocarcinoma cells, HCT116 (ATCC number CCL-247), and HCT116 Dicer negative cells (HCT116(-), a generous gift from Dr. Bert Vogelstein [45]) were maintained in DMEM supplemented with 10% FCS, 1000 U/mL P/S, 2% sodium bicarbonate, 1% sodium pyruvate and 1% MEM non-essential amino acids.

### IL-6 treatment

Cells were seeded on 6-well plates at a density of 5×10^4^ in growth media. After 24-hours of culture, the media was replaced with fresh growth media containing recombinant human IL-6 (Cat# PHC0064; Invitrogen, Carlsbad, CA, US) at the final concentration of 10 ng/ml every day. At the designated treatment time points, cells were harvested for genomic DNA and total RNA extraction.

### Demethylating treatment using 5 Aza-2′-deoxycitidine (5 Aza-dC)

HuCCT1 cells and HCT116 cells were plated on 6-well plates at a density of 5×10^4^ cells per well. After 24 hours of culture, media was replaced with fresh growth media containing 5 micromolar (µM) 5 Aza-dC (Cat# A3656; Sigma, Saint Louis, MO, US) every day . The media was changed to fresh 5 Aza-dC containing media every 24 hours. 0.001% Acetic Acid (Cat# BP1185500, Fisher Scientific, Pittsburgh, PA, US ) was used as vehicle control. At the designated treatment time points, cells were harvested and total RNA was extracted using Trizol reagent.

### Bisulfite sequencing

Bisulfite sequencing of IG-DMR-CG6, was carried out as described previously with some modifications [28]. Bisulfite conversion of genomic DNA was carried out using EpiTect Bisulfite Kit (Cat# 59104; QIAGEN Science, Germantown, MD, US ). PCR amplifications were performed in a 50 µL volume consisting of 100 ng genomic DNA, 50 mM of dNTPs, 1 µM of each forward and reverse PCR primer, 1 U of HotStar Taq DNA polymerase (Cat# 203203; QIAGEN Science, Germantown, MD, US ), and 1× PCR buffer. The PCR primer sequences were the following: 5′-GTTTATTGGGTTGGGTTTTGTTAG-3′ (IG-DMR-CG6 forward) and 5′-TAACCAATTACAATACCACAAAATTAC-3′ (IG-DMR-CG6 reverse). The thermal cycler program was as follows: an initial denaturation at 95°C for 15 minutes, 40 cycles of 95°C for 30 seconds, 59°C for 30 seconds, and 72°C for 30 seconds; and then a final extension at 5 minutes at 75°C. PCR products were cloned into PCR2.1 (the Original TA Cloning Kit (Cat# 45-0016; Invitrogen, Carlsbad, CA, US)). Seven to ten clones for each sample DNA were subjected to cycle sequencing on a 3730xl DNA Analyzer (Applied Biosystems, Carlsbad, CA, US) located in the Hopkins Sequencing Facility.

### Uniparental derived chromosome 12 (UPD(12)) disomy mouse generation

Paternally or maternally derived (pUPD(12) and mUPD(12), respectively) uniparental disomy mice were generated as described before [25]. Two e16.5 maternal disomy embryos and two e16.5 paternal disomy embryos and control littermates are used for this experiment. RNA was extracted from whole embryo and qRT-PCR was performed. Mature miR-370 sequence is conserved between human and mouse, thus we employed the same TaqMan miR Assay kits described above for the qRT-PCR.

### Analysis for allelic loss

We performed PCR on genomic DNA using Power SYBR Green PCR Master Mix (Cat# 4367659; Applied Biosystems, Carlsbad, CA, US). Genomic LINE-1 (Long Interspersed Nuclear Element type 1, or L1) DNA was used as control. The primers for LINE1 were the following: 5′-TGAACAACCTGCTCCTGAATGAC-3′ (forward) and 5′-TCCCAGAGATT CTGGTATGTGGT-3′ (reverse). The primers for miR-370 were the following: 5′-GGCGA ATTCTGCATTCCTCATTCTAC-3′ (forward) and 5′-GCGCTCGAGTAACGTCTCTGTG CCTG-3′ (reverse). The thermal cycler program was as follows: initial denaturation at 95°C for 10 minutes, 50 cycles at 95°C for 20 seconds, 56°C for 30 seconds, and 72°C for 30 seconds; and then a final extension for 5 minutes at 75°C. PCR reaction was carried out on the 7900 HT Fast Real-time PCR System (Applied Biosystems, Carlsbad, CA, US) in duplicate.

### Transfection with miR-370 mimic or inhibitor

The synthesized miR-370 mimic (miR-370M, Cat# C-300676-05), inhibitor (miR-370In, Cat# I-300166-01), Non-specific mimic (NSM, Cat# CN-001000-01-10) and non-specific-inhibitor (NSI, Cat# IN-001000-01-05) were purchased from Dharmacon (Lafayette, LA, US). 50∼70% confluent cells were transfected with 20 nM of miR-370M or miR-370In using Lipofectamine RNAi MAX (Cat# 13778-150; Invitrogen, Carlsbad, CA, US). NSM or NSI was used as negative control respectively. RNA and proteins were harvested 48 and 72 hours after transfection, respectively.

### Retroviral vectors, viral supernatant production and viral transduction

MSCV-based bicistronic retroviral vectors, MIEG3 (Ghiaur, 2006 #475) were used to express miR-370. The genomic DNA sequence of miR-370 was amplified using PCR primers flanked by EcoRI (5′) and XhoI (3′) and cloned into the multiple cloning site of MIEG3. The primers for genomic miR-370 were the the same as describe above. The expression of miR-370 was linked with expression of enhanced green fluorescence protein (eGFP) via internal ribosome entry site 2 (IRES2). The plasmid DNA was used to generate viral supernatant from 293-T cells as previously described (Wahlers, 2001 #519). Briefly, 293-T cells were grown to 70% confluence in a T75 tissue culture treated flask (Corning, Inc., Corning, NY, US). 8 micrograms (µg) of plasmid DNA of miR-370 together with 10 µg MLV gag-pol plasmid and 3 µg VSVG envelope plasmid were co-transfected using Lipofectamine 2000 (Cat# 11668-019; Invitrogen, Carlsbad, CA, US). Eight milliliters (ml) of viral supernatant was collected every 24 hours and stored at −80°C until used. Next, 3×10^5^ HuCCT1 cells were incubated with 2 mL of viral supernatant containing 8 mg/mL of hexadimethrine bromide (Polybrene, Cat# 107689; Sigma-Aldrich, Milwaukee, WI, US). After 6–8 h, the viral supernatant was discarded and fresh DMEM was added. Two days after transduction, cells were harvested and sorted for eGFP expression using a fluorescence activated cell sorter (FACSVantage SE DiVa, Becton Dickinson, San Jose, CA, US).

### Cell growth assay

Ten thousand cells were plated in 24-well plates and transfected 24 hours later (Day 0) and counted every other day using a hemocytometer and an inverted-light microscope.

### Luciferase reporter assay

#### PGL4 luciferase plasmid construction

According to *TargetScan* (http://www.targetscan.org/), miR-370 has a binding site in WNT10B 3′-untranslated region (3′UTR). A portion of the WNT10B 3′UTR, containing miR-370 predicted binding site (wild type), was amplified using linker primers containing XbaI restriction sites. Primers for the wild type were the following: 5′-GCGTCTAGAGAGAGTTGTCTGTCCAGG-3′ (forward) and 5′-GCGTCTAGAACAGG AACCAAGAACAAG-3′ (reverse). Furthermore, we searched for additional miR-370 binding sites in the 5′-untranslated region (5′UTR) as well as in the open reading frame (ORF) region and found another seven nucleotide putative binding site in the WNT10B ORF. A portion of the WNT10B ORF, containing miR-370 predicted binding site (wild type), was amplified using linker primers containing XbaI restriction sites. Primers for the wild type were the following:5′- GCGTCTAGATGGTGAGCTGTGGCTGTG-3′ (forward) and 5′-GCGTCTAG ATGTCCAT GTCATGGTTAC-3′ (reverse). Both amplicons were cut by XbaI and cloned into an XbaI site just downstream of the firefly luciferase structural gene in vector pGL4 (Cat# E6651; Promega, Madison, WI, US). For the 3′UTR mutant, the miR-370 binding site was mutated by substituting the eight nucleotides of the miR-370 binding sites using the Gene Tailor site directed mutagenesis system (Cat# 4500239; Invitrogen, Carlsbad, CA, US). Primers for the mutations were the following: 5′- CTCTTTTTCCTTCTTACA GCAACAGG-3′ (forward) and 5′- AAGAAGGAAAAAGAGG CTCCAAGAG-3′ (reverse). For the ORF mutant, the miR-370 binding site was mutated by substituting seven nucleotides of the miR-370 binding sites using the Gene Tailor site directed mutagenesis system. Primers for the ORF mutations were the following: 5′-GGAAGGGCAGTGGTGCTGGTTCTCG-3′ (forward) and 5′-CACCACTGCCCTTCCAGCCACAGCCA-3′ (reverse). All plasmids (wild-type and mutant) were verified by sequencing. After sequence verification, we obtained plasmid clones containing correctly oriented inserts. Six thousand cells per well were seeded onto 96-well plates 24 hrs prior to transfection. Cells were transfected with miR-370M or the NSM . 24 hrs after transfection the cells were co-transfected with constructed wild type or mutated pGL4 vector (firefly luciferase) and internal control pRL-CMV (Renilla luciferase, Cat# E2261; Promega, Madison, WI, US) vector. 48 hours after plasmid vector transfection, the luciferase reporter assay was performed using a Dual-Luciferase Reporter Assay System (Cat# P1041; Promega, Madison, WI, US). After 48 hrs, luminescence intensity was measured by Veritas Microplate Luminometer (Turner Biosystems, Madison, WI, US), and the luminescence intensity of firefly luciferase was normalized to that of Renilla luciferase.

### Western blotting

Cells were lysed in Laemmli sample buffer (Cat#161-0737; Bio-Rad, Hercules, CA) supplemented with a protease inhibitor (Complete, EDTA-free, Roche, Indianapolis, IN, US). Protein concentration was measured using a BCA Protein Assay kit (Cat# 23227; Thermo Scientific, Rockford, IL, US). Cell lysates (40–45 µg per lane) were electrophoresed on 10–20% polyacrylamide gels (Cat# 456-1084; Bio-Rad, Hercules, CA, US) and transferred to Immobilon-PSQ membranes (Millipore, Bedford, MA). The membranes were blocked with TBS containing 5% skim milk and 0.1% Tween-20 (TBST), then incubated with the primary antibody. Antibody to WNT10B was purchased from Abcam (Cat# ab91201; Abcam, Cambridge, MA, US). The membranes were incubated after TBST washing with HRP-conjugated anti-mouse secondary antibody, (Cat# 626520; Invitrogen, Frederick, MD, US) and analyzed using enhanced chemiluminescent HRP Antibody Detect Reagent (Cat# E2400; Denvillle Scientific, Inc, Metuchen, NJ, US).

### Statistical analysis

All data are presented as means ± SD. Student's t-test and one-way ANOVA were performed for comparing continuous variables of two- and three or more groups, respectively. Pearson correlation test was used for the evaluation of correlation between two continuous variables. Differences between group means with P values<0.05 were regarded as being statistically significant.

## Supporting Information

Figure S1 **DNA demethylation upregulates miR-370 expression in HCT116 cells.** Black column -5Aza-dC, gray column –negative control. X-axis –the time post 5Aza-dC treatment (hours). Y-axis –qRT-PCR expression of miR-370 *vs.* RNU6B. Data represents the mean value of three independent experiments. Mean ± SD. ***P<0.001.
(TIF)

Figure S2 **HCT116(-) cells display decreased growth upon miR-370 reinforced expression.** X-axis –HCT116(-) cells counted at day 2, 4, 6 and 8 after transfection of miR-370 mimic. Y-axis – counts ×10^4^ of HCT116(-) cells transfected with miR-370M (squares) or the control non-specific mimic (NSM - circles). Data represents the mean value of five independent experiments. Mean ± SD. **P<0.01.
(TIF)

Figure S3 **Wnt10b is downregulated at mRNA level by miR-370.** Y-axis – level of wnt10b in HuCCT1-EV cells (control cells) and in HuCCT1-370V cells (cells overexpressing miR-370). The level of wnt10b in HuCCT1-370V is approximately 60% of the level in control cells. Values were normalized to beta-actin.
(TIF)

Figure S4 **miR-370 is upregulated to physiologic levels through retrovirus-mediated delivery in contrast to transfections.** A. miR-370 is upregulated approximately 24,000 fold through transfections. non-specific mimic – NSM, miR-370 mimic - miR-370M. B. miR-370 is upregulated approximately 7.5-fold when retrovirus-mediated miR-370 delivery is employed. MIEG3 empty virus – EV, MIEG3-miR-370 virus - miR-370V. C. miR-370 is upregulated approximately 9.5 times in normal cholangiocytes (H69 cells) *vs.* malignant cholangiocytes (HuCCT1 cells)
(TIF)
