# Supplementary figures and images for: Silencing of miR-370 in Human Cholangiocarcinoma by Allelic Loss and Interleukin-6 Induced Maternal to Paternal Epigenotype Switch
Source: PLoS One. 2012 Oct 22;7(10):e45606. doi: 10.1371/journal.pone.0045606 (PMC3478287; doi:10.1371/journal.pone.0045606)

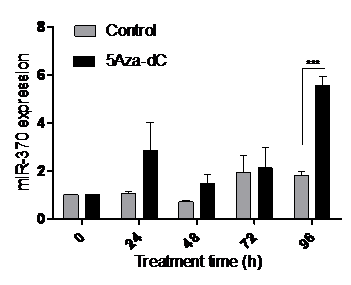

Supplement: Figure S1 — DNA demethylation upregulates miR-370 expression in HCT116 cells. Black column -5Aza-dC, gray column –negative control. X-axis –the time post 5Aza-dC treatment (hours). Y-axis –qRT-PCR expression of miR-370 vs. RNU6B. Data represents the mean value of three independent experiments. Mean ± SD. ***P<0.001. (TIF) [file pone.0045606.s001.tif]

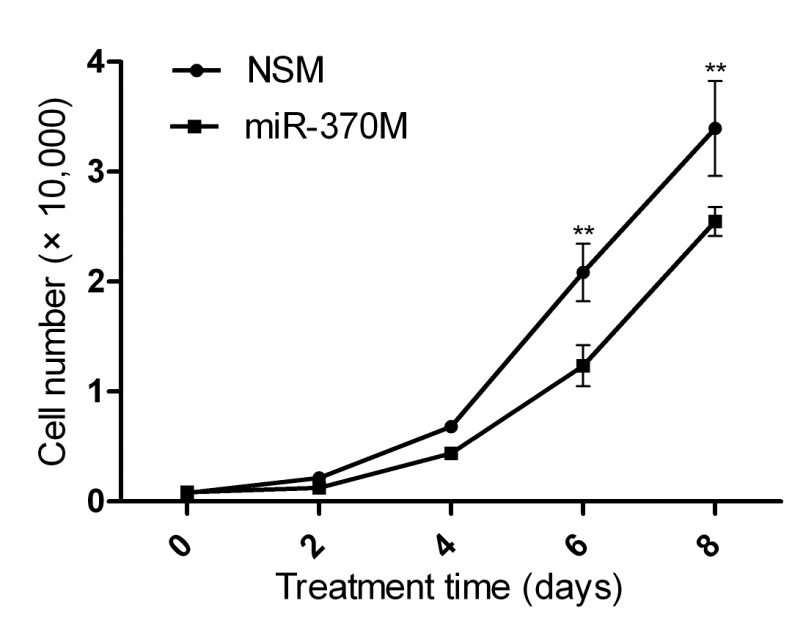

Supplement: Figure S2 — HCT116(-) cells display decreased growth upon miR-370 reinforced expression. X-axis –HCT116(-) cells counted at day 2, 4, 6 and 8 after transfection of miR-370 mimic. Y-axis – counts ×104 of HCT116(-) cells transfected with miR-370M (squares) or the control non-specific mimic (NSM - circles). Data represents the mean value of five independent experiments. Mean ± SD. **P<0.01. (TIF) [file pone.0045606.s002.tif]

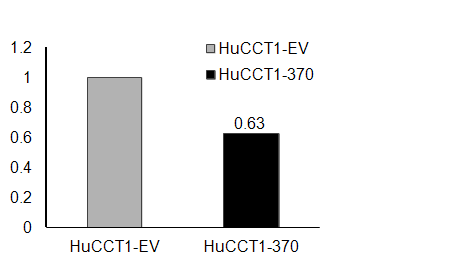

Supplement: Figure S3 — Wnt10b is downregulated at mRNA level by miR-370. Y-axis – level of wnt10b in HuCCT1-EV cells (control cells) and in HuCCT1-370V cells (cells overexpressing miR-370). The level of wnt10b in HuCCT1-370V is approximately 60% of the level in control cells. Values were normalized to beta-actin. (TIF) [file pone.0045606.s003.tif]

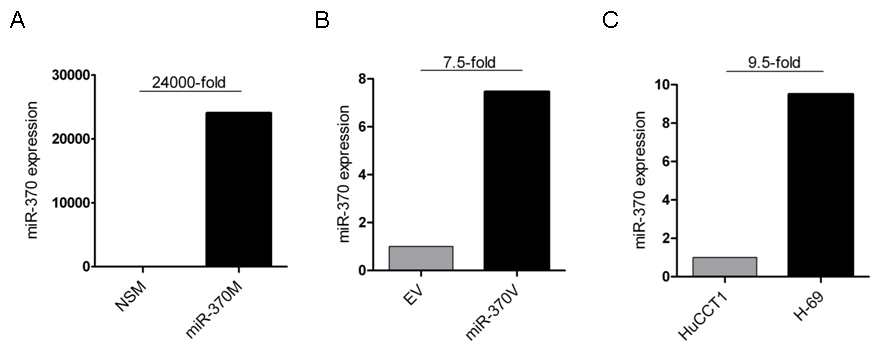

Supplement: Figure S4 — miR-370 is upregulated to physiologic levels through retrovirus-mediated delivery in contrast to transfections. A. miR-370 is upregulated approximately 24,000 fold through transfections. non-specific mimic – NSM, miR-370 mimic - miR-370M. B. miR-370 is upregulated approximately 7.5-fold when retrovirus-mediated miR-370 delivery is employed. MIEG3 empty virus – EV, MIEG3-miR-370 virus - miR-370V. C. miR-370 is upregulated approximately 9.5 times in normal cholangiocytes (H69 cells) vs. malignant cholangiocytes (HuCCT1 cells) (TIF) [file pone.0045606.s004.tif]
